# Anti- and Pro-Lipase Activity of Selected Medicinal, Herbal and Aquatic Plants, and Structure Elucidation of an Anti-Lipase Compound

**DOI:** 10.3390/molecules181214651

**Published:** 2013-11-26

**Authors:** Muhammad Abubakar Ado, Faridah Abas, Abdulkarim Sabo Mohammed, Hasanah M. Ghazali

**Affiliations:** 1 Department of Food Science, Faculty of Food Science and Technology, University Putra Malaysia UPM, Serdang 43400, Selangor, Malaysia; 2 Institute of Biosciences, University Putra Malaysia UPM, Serdang 43400, Selangor, Malaysia

**Keywords:** anti-lipase activity, pro-lipase activity, plant extracts, orlistat equivalent, kaempferol 3-*O*-rhamnoside

## Abstract

Plants that help in slowing down the digestion of triacylglycerols (TAGs) in the pancreas and small intestine of humans play an important role in the reduction of obesity. On the other hand, there may be plants or plant parts that stimulate intestinal lipolytic activity, thus contributing to greater TAG assimilation. The aim of this study was to evaluate the aqueous methanolic extracts of ninety eight (98) medicinal, herbal and aquatic plant materials from Malaysia for their effect on porcine pancreatic lipase (PPL) activity and to identify the structure of an anti-lipase compound from one of the sources. The degree of inhibition was also quantified as relative to orlistat activity against PPL (orlistat equivalents). Results revealed that while 19.4% of the extracts were found to have anti-lipase activity ≥80%, 12% were actually found to promote PPL activity. Twenty two percent (22.4%) exhibited moderate inhibition (41%–80%) and 2% were neutral toward PPL activity. The ripe fruit of *Averrhoa carambola* and the leaves of *Archidendron jiringa* (Jack) I.C Nielsen L. (*jering*), *Cynometra cauliflora* (*nam-nam*) and *Aleurites moluccana* (L.) Willd (candle nut/*buah keras*) had the highest (100%) anti-lipase activity and are equivalent to 0.11 µg orlistat/mL. Plants that stimulated lipase activity included *Pimpinella anisum* L. (aniseed/*jintan manis*), activating the enzyme by 186.5%. Kaempferol 3-*O*-rhamnoside was isolated from the ethyl acetate fraction of *C. cauliflora* leaves and found to be an active lipase inhibitor. The structure was elucidated using ^1^H-NMR, ^13^C-NMR and 2D-NMR analyses.

## 1. Introduction

Obesity is defined in terms of body mass index (BMI), which is calculated as body weight divided by square of height, and it is mainly a result of an imbalance between energy intake and expenditure [1]. The World Health Organization (WHO) has reported that worldwide obesity has more than doubled since 1980 [2]. In 2008, more than 1.4 billion adults aged 20 or older were overweight, with women outnumbering men by 3:2. More than 40 million children under the age of five were overweight in 2010 and in 2012 overweight and obesity represented the fifth leading risk for global deaths, with at least 2.8 million adult deaths. Many diseases such as hypertension, non-insulin dependent hyperlipidemia, and diabetes mellitus and coronary heart diseases are attributable to overweight and obesity [2]. In Malaysia, some of the diseases related to obesity are increasing at a rate to such an extent that cardiovascular disease is now the fundamental cause of death [3].

Overweight and obesity were once considered a high-income country problem, but now these conditions are on the rise in low- and middle-income countries, particularly in urban settings [4,5]. It has been reported that among Malaysians, one in four is overweight or obese and the percentage of obese Malaysians is double what it was a decade ago [5]. A survey of more than 50,000 school children in Malaysia confirmed that a higher percentage of children in urban areas are obese compared to those in rural areas [6]. Although there appear to be a decline in the rate of increase or even a leveling off in prevalence of obesity, 35% of men and women in the USA were still considered as obese in 2009–2010 with no significant difference in prevalence between men and women at any age [7].

Lipases (triacylglycerol hydrolase E.C. 3.1.1.3) are enzymes that catalyze the hydrolysis of ester bonds of triacylglycerols (fats and oils) to produce free fatty acids, diacylglycerols, monoglycerols and glycerol. In the small intestine of mammals, the digestion of dietary triacylglycerols (TAG) is essentially due to the action of pancreatic lipase. The end products after they have been absorbed by the body are responsible for the development of obesity. Therefore, if the hydrolysis of TAG, and thus, its movement from the intestinal lumen into the body is stopped or minimized, the prevalence of obesity can be reduced [8,9]. For this reason, an inhibitor of digestive lipases could become a useful anti-obesity agent.

One of the approaches to reduce obesity is treatment with synthetic drugs such as sibutramine, rimonabant, phentermine, diethylpropion, zonisamide, topiramate and orlistat [10]. Orlistat (N-formyl-L-leucine (1*S*)-1-[(2*S*,3*S*)-3-hexyl-4-oxetanyl]methyldodecyl ester, known also as tetrahydrolipstatin) is a unique non-centrally acting anti-obesity compound that acts within the gastrointestinal tract by impeding pancreatic and gastric lipases that play an essential role in the digestion of long chain TAG. At the recommended therapeutic dose of 120 mg three times a day, it inhibits dietary fat absorption by about 30% [11,12]. It is also the only anti-obesity drug that is approved for long term weight management [10]. Sibutramine and rimonabant were recently withdrawn from the market due to adverse side effects. Sibutramine could lead to an increased risk of heart attack and stroke in high-risk cardiac patients, whereas the latter could lead to potentially serious psychiatric disorders. Hence, there is an urgent need for safer and more efficient anti-obesity agents from natural sources, even though they are also toxic, but are of less damaging as compared with the pure synthetic ones [13].

There have been several reports on the search for anti-lipase inhibitors from natural sources [14,15,16,17,18,19,20]. Although to date there are no reports on plants with pro-lipase activity, it is possible that some plants may also possess metabolites that will stimulate the activity of pancreatic lipases. Thus, the objective of this study was to examine the crude methanol extracts of ninety eight (98) different parts (seeds, fruits, leaves, stems, flowers, roots) of some medicinal, herbal and aquatic plants largely found in Malaysia for their anti-lipase, pro-lipase activity as well as to identify an anti-lipase compound from one of the plant sources.

## 2. Results and Discussion

### 2.1. Effect of Methanol Extract of Medicinal, Herbs, and Aquatic Plants on Porcine Pancreatic Lipase

**Figure 1:**
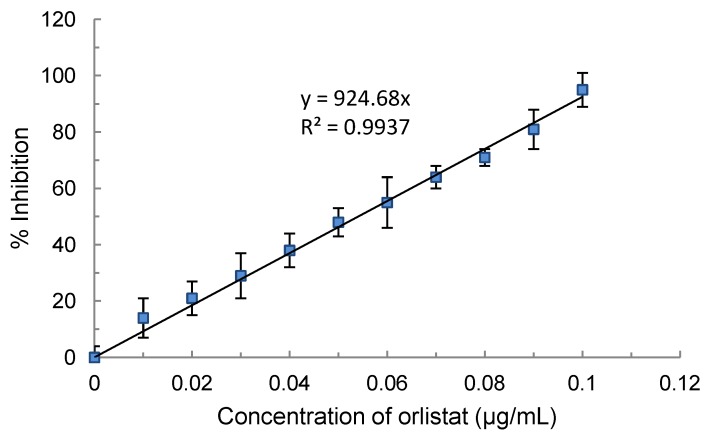
Inhibitory effect of orlistat on porcine pancreatic lipase.

**Figure 2:**
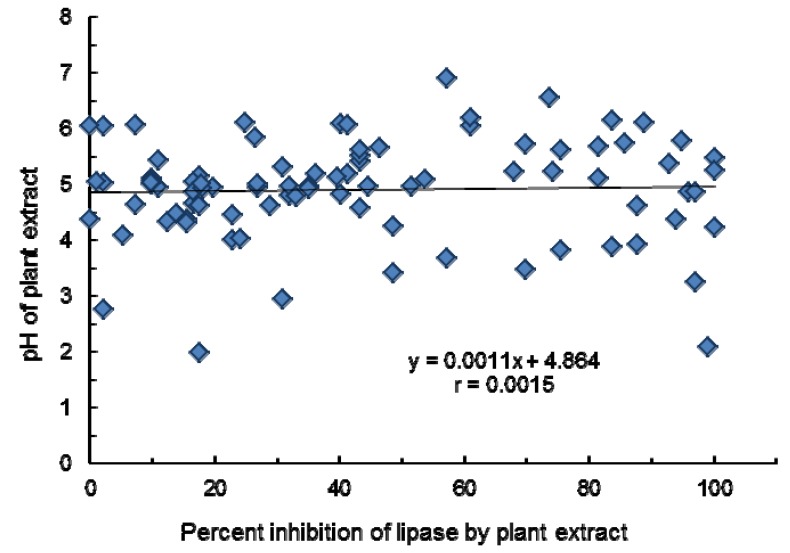
Correlation between pH of extract and percent lipase inhibition.

The methanol extracts of ninety eight (98, sixty leaves, twelve fruits, four seeds, fifteen whole plants, two stems, one flower, and four roots) were screened for their effect against porcine pancreatic lipase (PPL) activity. The results obtained are tabulated to show the list of plants that inhibit lipase activities, equivalent and pH of the extracts in (Table 1) and those that stimulated (Table 2) the activity of enzyme. The anti-lipase activities are also expressed as percent (%) inhibition relative to that of orlistat (*i.e.*, orlistat equivalents) as this drug also inhibits pancreatic lipase activity in the duodenum. The inhibitory curve for orlistat is shown in Figure 1, where 0.1 µg/mL of orlistat resulted in 95% inhibition of pancreatic lipase activity. As there is a likelihood that the inhibitory or stimulatory effect observed could be due to the pH of the plant extracts, a plot between pH and percent inhibition was constructed (Figure 2). From the figure, it can be concluded that there is no correlation between the pH of the extract on lipase inhibition, and thus the inactivation was not most likely not due to the pH of the extract.

### 2.2. Isolation of Lipase Inhibitory Compound Using Bio-Assay Guided Isolation Protocol

#### 2.2.1. Fractionation of Extract Residue of the Active *C. cauliflora* L. Leaves

**Figure 3:**
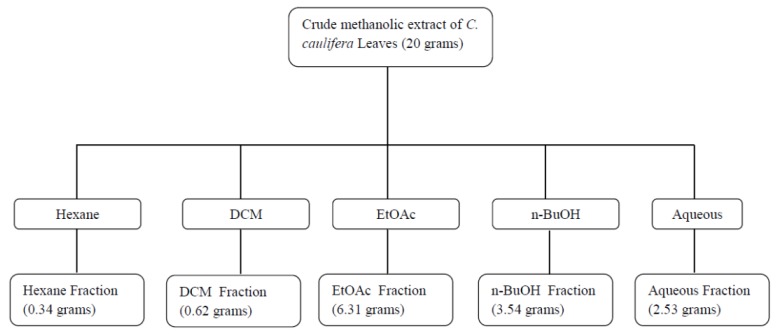
Fractionation scheme of crude methanol extract of *C. caulifera* leaves.

The methanolic residue (20 g) from 1 kg of ground oven-dried leaves of *C. cauliflora* L. was dissolved in 200 mL water/methanol (1:3) and fractionated using the scheme shown in Figure 3. First, the extract was partitioned four times with a total volume of 800 mL of *n*-hexane. The *n*-hexane layers were collected and evaporated to yield the *n*-hexane fraction (0.34 grams). The aqueous fraction was further subjected to successive solvent-solvent extraction using first dichloromethane (DCM), followed by ethyl acetate (EtOAc) and finally with *n*-butanol (*n*-BuOH). The same procedure used to obtain the *n*-hexane extract was applied. The process resulted in 0.62 grams, 6.31 grams and 3.54 grams of DCM, EtOAc and n-BuOH fractions. The remaining aqueous extract was evaporated under reduced pressure to dryness to give 2.53 grams of water fraction. Each fraction was then subjected to the inhibition test against lipase activity.

**Table 1:** Lipase inhibitory activity of plant extracts.

No.	Scientific Name	Local Name	Part Used	Family	Percent Inhibition	Orlistat Equiv. (µg/mL)	pH of Extract
1	*Adenanthera bicolor* Moon	Daun tajam	L	Acanthaceae	26.4	0.03	5.84
2	*Adenanthera bicolor* Moon	Daun tajam	SD	Acanthaceae	26.8	0.03	4.95
3	*Aleurites moluccana* L. Willd	Buah keras (candle nut)	L	Euphocynacea	100.0	0.11	5.47
4	*Allamanda cathartica* L.	Alamanda	F	Aphobiaceae	0.0	0.00	4.38
5	*Allium cepa* L.	Bawang merah (shallot)	RT	Liliaceae	73.5	0.08	6.56
6	*Allium sativum*	Bawang putih (garlic)	RT	Apiaceae	44.6	0.05	4.96
7	*Alocasia macrorrhizos* L. (G) Don	Keladi ganyong (yam)	L	Araceae	12.4	0.01	4.34
8	*Amomun cardamomum* L. Maton	Pelaga (cardamom)	SD	Zingiberaceae	36.1	0.04	5.18
9	*Anacardium occidentale* L*.*	Gajus (cashew)	L	Anacardiaceae	88.7	0.10	6.11
10	*Andrographi panicutata* (Burmf.)Wall	Hempedu bumi	L	Acanthacea	69.8	0.08	3.47
11	*Archidendron jiringa* (Jack) I.CNielsen	Jering	F	Falaceae	100.0	0.11	5.25
12	*Asystasia gangetica* L. T. Anderson	Akar ruas-ruas (Chinese violet)	L	Acanthaceae	17.5	0.02	5.15
13	*Averrhoa carambola* L*.*	Belimbing besi (starfruit)	FR	Oxalidaceae	100.0	0.11	4.23
14	*Averrhoa carambola* L*.*	Belimbing besi (starfruit)	L	Oxalidaceae	94.8	0.10	5.78
15	*Azadirachta indica* A. Juss	Semambu (neem)	L	Meliaceae	93.8	0.10	4.38
16	*Barringtononia racemaso* L. Spreng	Putat kampong	L	Lecythidaceae	95.9	0.10	4.86
17	*Brassica oleracea* L.	Kobis (cabbage)	L	Brassicaceae	46.4	0.05	5.67
18	*Brassica* sp.	Sawi hijau	WP	Brassicaceae	7.2	0.01	6.04
19	*Citrus hystrix* DC.	Limau purut	L	Rutaceae	1.0	0.00	4.45
20	*Coleus amboinicus* Lour.	Bangun-bangun	L	Labiateae	9.6	0.01	4.83
21	*Colubrina asiatica* L. Brongn	Peria pantai	L	Rhamnaceae	10.3	0.01	4.81
22	*Coriandrum sativum* L.	Ketumbar (coriander)	SD	Apiaceae	16.5	0.02	5.03
23	*Cosmos caudatus* Kunth	Ulam raja	WP	Asteraceae	35.1	0.04	4.25
24	*Cuminum cyminum* L*.*	Jintan putih (cumin)	SD	Apiaceae	44.6	0.05	5.41
25	*Curcuma longa* L.	Kunyit (tumeric)	RT	Zingerberaceae	62.9	0.07	4.95
26	*Curcuma longa* L.	Kunyit (tumeric)	L	Zingerberaceae	52.7	0.06	3.24
27	*Curcuma xanthorrhiza* Roxb	Temu lawak	L	Zingerberaceae	16.9	0.02	4.62
28	*Cynometra cauliflora* L.	Nam-nam	L	Falaceae	100.0	0.11	4.65
29	*Cynometra cauliflora* L.	Nam-nam	FR	Falaceae	97.9	0.11	3.88
30	*Emblica officinalis*	Melaka	L	Euphobiceae	76.3	0.08	5.51
31	*Euginia michelii* L*.*	Cermai belanda	ST	Mytaceae	90.7	0.10	4.97
32	*Euginia michelii* L*.*	Cermai belanda	L	Mytaceae	92.8	0.10	4.38
33	*Ficus deltoidea* Jack*var. kunstleri* (King Corner)	Mas cotek	FR	Moraceae	22.7	0.02	3.83
34	*Hibiscus esculentus* L*.*	Kacang bendi (okra)	L	Malvaceae	10.3	0.01	5.20
35	*Hibiscus sabdariffa* L.	Asam susur (roselle)	L	Malvaceae	60.9	0.07	6.20
36	*Illicium verum hook* fil	Bunga lawang (star anise)	SD	Illiciaceae	22.7	0.02	4.00
37	*Ipomonea reptans* Forsk	Kangkung (water convolvulus)	L	Convolvulaceae	40.2	0.04	6.09
38	*Labisia* sp.	Halia bara (red ginger)	RT	Myrsinaceae	32.0	0.03	4.97
39	*Lawasonia inermis* L.	Inai (henna)	L	Lythraceae	2.1	0.00	2.75
40	*Leea indica*	Memali	L	Leeaceae	48.5	0.05	3.41
41	*Leucaena leucocephala* (Lam) Dewit	Petai belalang	L	Fabaceae	43.3	0.05	4.58
42	*Limnocharis flava* L. Buchenau	Emparut	L	Limnocha-ritaceae	10.9	0.01	5.43
43	*Melastoma melabathricum*	Senduduk	L	Melastomataceae	96.9	0.10	4.86
44	*Melastoma melabathricum*	Senduduk	FR	Melastomataceae	87.6	0.09	3.93
45	*Mentha piperita* L. (Pro. Sp)	Pudina (mint)	L	Lamiaceae	16.5	0.02	5.04
46	*Momordica charantia* L.	Peria katak	FR	Cucurbitaceae	83.6	0.09	6.14
47	*Monochoria hastata* L. Solms	Keladi agas	L	Pontederiaceae	43.3	0.05	5.61
48	*Morinda citrifolia* L.	Mengkudu (noni)	FR	Rubiceae	35.1	0.04	4.92
49	*Morinda citrifolia* L.	Mengkudu (noni)	L	Rubiceae	15.5	0.02	4.31
50	*Moringa oleifera* Lam	Kelor	L	Moringaceae	75.4	0.08	5.62
51	*Murraya koerugii* Spreng.	Kari	L	Rutaceae	41.2	0.04	6.07
52	*Musa acuminate* Colla	Pisang (banana)	L	Musaceae	16.5	0.02	4.87
53	*Musa acuminate* Colla	Jantung pisang	FR	Musaceae	19.6	0.02	4.95
54	*Nigella sativa* L*.*	Jintan hitam	SD	Ranunculaceae	37.1	0.04	5.12
55	*Oenanthe javanica* (Blume) DC*.*	Selom	L	Apiaceae	39.5	0.04	5.01
56	*Ormosia bancana* (miq.) Merr.	Saga dengkol	SD	Fabaceae	26.8	0.03	6.10
57	*Orthosiphon spicatus* BBS	Misai kucing	WP	Acanthaceae	24.8	0.03	5.23
58	*Pakia speciosa* Hassk	Petai	ST	Fabaceae	74.2	0.09	5.09
59	*Pakia speciosa* Hassk	Petai	P	Fabaceae	53.6	0.06	4.92
60	*Pakia speciosa* Hassk	Petai	L	Fabaceae	18.0	0.02	4.62
61	*Pandanus amaryllifolius*	Pandan	L	Pandanaceae	17.5	0.02	5.72
62	*Peperomia pellucid* L. Kunth	Ketumpangan air	WP	Piperales	69.8	0.08	4.64
63	*Pereskia sacharosa* Griseb	Jarum tujuh bilas	L	Cactaceae	7.2	0.01	5.37
64	*Persea americana* Mill	Avocado	L	Lauraceae	92.8	0.10	6.07
65	*Phyllanthus minima*	Letup-letup	L	Solanaceae	7.2	0.01	5.68
66	*Phyllanthus niruri* L*.*	Dukung anak	WP	Euphorbiaceae	81.4	0.09	5.11
67	*Piper battle* L*.*	Sirih	WP	Piperaceae	9.9	0.01	5.01
68	*Piper nigrum* L.	Lada putih (white pepper)	SD	Piperaceae	24.1	0.03	4.03
69	*Piper sarmentosum* Roxb	Kadok	L	Piperaceae	2.1	0.00	6.04
70	*Pisonia grandis* R.Br.	Mengkudu siam	L	Nyctaginaceae	1.0	0.00	5.05
71	*Pista statiotes* L*.*	Selada air	WP	Araceae	57.2	0.06	6.91
72	*Premma cordiflora*	Buas-buas	L	Verbenaceae	30.9	0.03	2.94
73	*Premma cordiflora*	Buas-buas	SD	Verbenaceae	13.9	0.02	4.48
74	*Premma cordiflora*	Buas-buas	RT	Verbenaceae	17.5	0.02	1.98
75	*Psidium guajava* L.	Jambu (guava)	L	Myrtaceae	99.0	0.11	2.09
76	*Saricocalix crispus*	Pecah kaca	L	Acanthaceae	30.9	0.03	5.31
77	*Sauropus androgynus* L. Merr.	Cekur manis	RT	Euphorbiaceae	9.9	0.01	5.06
78	*Solanum mammosum* L.	Terung susu kambing (nipplefruit)	L	Solanaceae	33.0	0.04	4.79
79	*Spermacoce latifolia* Aubl	Kutu tahang (buttonweed)	L	Rubiaceae	57.2	0.06	3.68
80	*Syzygium polyanthum*	Serai kayu/daun salam	L	Olacaceae	5.2	0.01	4.09
81	*Syzygium malaccense* L*.*	Jambu air	L	Myrtaceae	85.6	0.09	5.74
82	*Tamarindus indica* L*.*	Asam jawa (tamarind)	L	Fabaceae	9.9	0.01	5.03
83	*Tamarindus indica* L*.*	Asam jawa (tamarind)	FR	Fabaceae	68.0	0.07	5.24
84	*Tinospora crispa* (Miers)	Patawali	WP	Menispermaceae	28.9	0.03	4.62
85	*Trigonella foenumgraecum* L.	Halba (fenugreek)	SD	Fabales	0.0	0.00	6.04
86	*Vitex* sp.	Lemuni hitam	L	Verbenaceae	51.5	0.06	4.97

Plant Parts: F, Flowers; FR, Fruits; ST, Stem; SD, Seeds; P, Pod with seeds removed; WP, Whole plant. Results are presented as mean ± standard deviation. Mean was taken as the average of three readings of each experiment.

**Table 2:** Plant extracts that activate porcine pancreatic lipase.

No.	Scientific Name	Local Name	Part Used	Family	Percent Activation	pH of Extract
1	*Capsium frutescens* L.	Cili api	FR	Solanaceae	125.8	5.21
2	*Cymbopogen citrates* DC*.*	Serai (lemon grass)	WP	Graminae	104.6	6.04
3	*Emilia sonchifilia*	Tambak-tambak	WP	Asteraceae	131.6	3.10
4	*Gynura procumbens* (Lour) Merr	Sambung nyawa	WP	Compositae	120.6	6.87
5	*Kaempferia galanga* L.	Kencur (aromatic ginger)	RT	Zingiberaceae	122.3	5.47
6	*Lindernia crustacean* L*.*	Unknown	L	Scrophulariaceae	105.2	6.39
7	*Monochoria hastata* L. Solms	Keladi agas	RT	Pontederiaceae	103.1	6.37
8	*Ocimum basilicum* L*.*	Selasih (basil)	L	Lamiaceae	103.1	5.81
9	*Pimpinella anisum* L.	Jintan manis (aniseed)	SD	Apiaceae	186.5	6.41
10	*Plunchea indica* L. Less	Beluntas	L	Compositae	110.3	5.37
11	*Psophocarpus tetragonolobus* L.	Kacang botor (winged bean)	FR	Falacea	109.3	4.07
12	*Typha angustifolia* L*.*	Lembang	WP	Typhaceae	106.9	4.17

Plant Parts: FR, Fruits; SD, Seeds; WP, Whole plant. Results are presented as mean ± standard deviation. Mean was taken as the average of three readings of each experiment.

#### 2.2.2. Effect of *C. cauliflora* Fractions on Porcine Pancreatic Lipase

Lipase inhibitory activity test showed (Table 3) that the EtOAc fraction was the most potent (85.3%) fraction followed by the *n*-BuOH (71.3%) fraction. Less than (20%) inhibition was detected in the *n*-hexane and DCM fractions.

**Table 3:** Percent lipase inhibition of various fractions from *C. cauliflora* methanol extract.

Fraction	Percent Inhibition	Orlistat Equiv. (µg/mL)
Hexane fraction	14.8	1.84
DCM fraction	17.8	2.21
EtOAc fraction	85.3	10.59
*n*-BuOH fraction	71.5	8.88
Aqueous fraction	54.7	6.79

### 2.3. Column Chromatography and Isolation of Anti-Lipase Compound from EtOAc Fraction of *C. cauliflora* Leaves

The active EtOAc fraction of the plant was further subjected to a silica gel (Merck Kieselgel 60 Art No. 9385.1000, particle size 0.040–0.063 mm) column chromatography (11.6 × 6.5 cm diameter). The separation of compounds in the mixture was done by eluting first with ethyl acetate-chloroform followed by ethyl acetate-methanol of increasing polarity resulting in twenty (20) fractions of 100 mL each. Separation of compounds in the fractions was achieved by spotting the fractions on analytical TLC made from aluminum sheets coated with silica gel 60 F_254_ (Merck 1.05735, 40 mm × 80 mm × 0.25 mm) using pure methanol-water (1:1) as the mobile phase. TLC separation was conducted at room temperature (average 27 °C). Detection of compounds was made possible by spraying with 10% sulfuric acid solution followed by heating at 65 °C. Similar bands from TLC several plates were scrapped and pooled together after been evaporated under reduced pressure to produced six sub-fractions (A1–A6). Inhibitory test was also conducted on each of this sub-fraction (Table 4). As Sub-fraction A3 (51%) was found to be the most active, it (65 mg) was further subjected to Sephadex LH-20 chromatography (15.7 × 4.1 cm diameter) using pure methanol as the solvent to give four sub-fractions (A3-1, A3-2, A3-3, A3-4) of 5 mL each. Similarly, lipase inhibitory activity was assayed after each sub-fraction was dried. Sub-fraction A3-3 was found to be the most active (45.1%) (Table 5). The active A3-3 (45 mg) sub-fraction was further subjected to purification in a mini (7.5 cm height, and 1.5 cm diameter) Sephadex LH-20 chromatographic column by using pure methanol as the eluent to afford a dark orange amorphous colored powder after it has been dried using a rotary vacuum evaporator at 42 °C. The compound was detected as a single band by silica gel TLC after examination under UV light at short wavelengths (254 nm).

**Table 4:** Percent lipase inhibition of various sub-fractions from EtOAc fraction of *C. cauliflora*.

Sub-Fraction	Percent Inhibition	Orlistat Equiv. (µg/mL)
A1	31.5	3.91
A2	26.1	3.24
A3	51.8	6.43
A4	23.5	2.91
A5	13.9	1.72
A6	36.1	4.48

**Table 5:** Percent lipase inhibition of various fractions from Sub-fraction A3 of *C. cauliflora*.

Sub-Fraction	Percent Inhibition	Orlistat Equiv. (µg/mL)
A3-1	11.4	1.42
A3-2	27.5	3.41
A3-3	45.1	5.59
A4-4	13.2	1.64
A3-1	11.4	1.42
A3-2	27.5	3.41

### 2.4. HPLC Profiling of Compounds from Sub-Fractions A3 and A3-3

**Figure 4:**
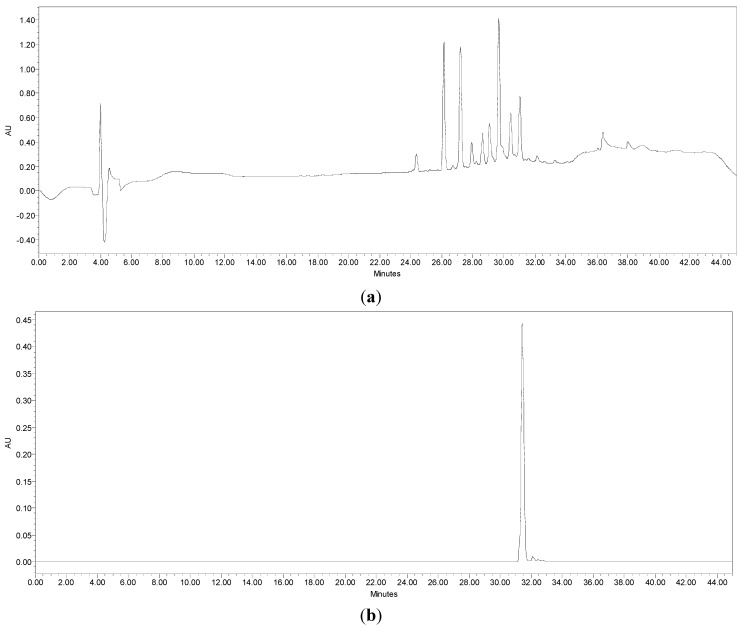
HPLC profiles of compounds from (a) Sub-fraction A3 from EtOAc fraction and (b) Sub-fraction A3-3 of *C. cauliflora* leaves extract.

The HPLC analysis was achieved by the use of a Waters HPLC system which is equipped with a Waters HPLC 600 pumping system Waters 2487 Dual λ absorbance detector and Waters Integrated software. A Jones (Darmstadt, Germany) chromatographic C_18_ reverse phase (particle size 5 µm) column (250 × 4.6 mm) placed in a column oven at 35 °C was used for the separation process. The column was equilibrated prior to the injection of each sample by flushing with the mobile phase solvents comprising 1% formic acid in HPLC grade acetonitrile for about 5 min followed by 1% formic acid in Miliq water (7:3) v/v. The active Sub-fraction A3, and the active compound isolated from Sub-fraction A3-3 were injected (10 μL) separately into the HPLC to obtain the profiles shown in Figure 4a,b, respectively.

### 2.5. Structure Elucidation of Anti-Lipase Compound

Identification of Anti-Lipase Compound from *C. cauliflora*

The powdered leaves of *C. cauliflora* were sequentially extracted at room temperature with 80% methanol, followed by a number of chromatographic steps were involved together with enzymatic activity test. The result of these processes confirmed that kaemferol*-3-O-*rhamnoside to be an active lipase inhibitor. It was purified as a yellow amorphous solid. The UV spectrum of the compound showed maximum absorption at 235.0, 270.0, and 335.0 nm. The HSQC data analysis (Table 6) was used to identify the respective positions of all protonated carbon atoms in the compound. 

The ^1^H-NMR spectral data indicated the existence of two aromatic protons at δ 7.77, and δ 6.94 (2H, *d*, *J* = 8.5 Hz) and (2H, *d*, *J* = 9.0 Hz), respectively, and with three protons on the methylene group of the rhamnose δ 0.98 (3H, *d*, *J* = 5.5 Hz) at position C-6". In the same way, singlet signals at δ 6.37, 6.20, and 4.22 were assigned to positions C-8, C-6 and C-2 of the kaempferol moiety. Signals of the other three protons of the compound, *i.e.*, δ 3.71 (1H, *t*, *J* = 8.5 Hz), δ3.34 (1H, *t*, *J* = 7.0 Hz), and δ 3.31 (1H, *t*, *J* = 3.0 Hz) were allocated at positions C-3", C-4", and C-5" respectively, with anomeric proton δ 5.39 at position C-1" of the rhamnose. However, all of the twenty one (21) carbon atoms in compound were leveled based on the comparison with literature values [21].

**Table 6:** ^1^H-NMR and ^13^C-NMR (500 MHz, CD_3_OD) assignment of kaempferol-*3*-*O-*rhamnoside. 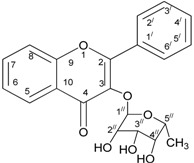

Position	δH	δC Reported Values	δC Experimental Values	COSY
1	-	-	-	-
2		155.7	157.5	
3		134.6	135.0	
4		177.9	178.4	
5		160.9	160.4	
6	6.20	99.3	98.8	
7		161.2	164.9	
8	6.37	94.4	93.6	
9		155.9	158.0	
10		104.9	103.0	
1'		120.4	121.5	
2'	7.77	130.6	130.7	7.77, 6.95
3'	3.31	115.3	115.4	7.77, 6.95
4'		160.0	162.1	
5'	6.94	115.3	115.6	7.77, 6.95
6'	7.77	130.6	130.7	7.77, 6.95
Rhamnoside C-3				
1"	5.39	101.9	102.3	5.39, 4.22
2"	4.22	70.1	70.7	
3"	3.72	70.6	70.9	3.72, 4.22
4"	3.34	73.8	72.0	
5"	3.32	70.2	70.9	3.32, 3.34
6"	0.98	17.8	16.5	

The HMBC correlations clearly indicated the attachment of the aromatic proton of the rhamnose at position C-1" (Table 7). The Heteronuclear Multiple Quantum Coherence (HMBC) spectrum of the compound illustrated all the possible locations/places of the carbon atoms relative to the protons in the compound. For example, positions C-7 and C-3 were confirmed by comparing the relationship between the hydrogen at position C-8 and C-3 with C-1", respectively. Positions C-8 and C-9 was identified with the protons at C-6 and C-8 and C-2' in that order. Position C-10 was located by relating the position of hydrogen at C-8 and C-6. Hydrogen located at C-5', C-6' were used to identified the place of C-1' and C-2'. Moreover, hydrogen located at C2' and C-5' were used to determine the position of C-3'. Also, C-4' was placed with hydrogen at positions C-5' and C-6. Position C-5' was located by using the proton on C-3' [21].

**Table 7:** HMBC correlations of protons relative to the carbon atoms. 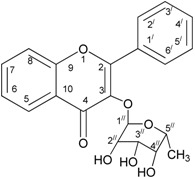

Position	Number of Correlated Proton δ H with C	Reported Values δC	Experimental Values δC
1	-	-	-
2		155.7	157.5
3	5.39	134.6	135.0
4	-	177.9	178.4
5	6.95, 7.77	160.9	160.4
6	6.37	99.3	98.8
7	6.37, 6.20	161.2	164.9
8	6.02	94.4	93.6
9	6.37, 7.77	155.9	158.0
10	6.37, 6.20	104.9	103.0
1'	6.95	120.4	121.5
2'	7.77	130.6	130.7
3'	6.95, 7.77	115.3	115.4
4'	6.95, 6.20	160.0	162.1
5'	6.95	115.3	115.6
6'	-	130.6	130.7

## 3. Discussion

As can be observed, plant parts that possessed inhibitory activity against PPL far outnumbered those that stimulated its activity. The ripe fruits of *A. carambola* and the leaves of *A. jiringa* (Jack) I.C *Nielsen L.*, *C. cauliflora*, and *A. moluccana* (L.) Willd were found to completely inhibit PPL activity, expressed as orlistat equivalents. These plants have a lipase inhibitory value equivalent to 12.4 µg orlistat/mL. Another 15 extracts reduced the activity of the enzyme by more than 80% and these may also be regarded as potential and useful sources of anti-obesity agents. Only 2% did not possess any inhibitory activity. Interestingly, almost similar degrees of inhibition were observed when comparing extracts from different parts of the same plant. For example, there is no difference in the degree of inhibition between the leaves and seeds of *A. bicolor* Moon. Similar levels of inhibition of PPL were observed between the extracts of the ripe fruits and leaves of *A. carambola* L., the ripe fruits and leaves of *C. cauliflora*, the roots and leaves of *C. longa* L., the stems and leaves of *E. michelii* L*.*, the fruits and leaves of *M. citrifolia* L., as well as the leaves, seeds and stems of *P. cordiflora*. However, there are also some variations in the inhibitory activity of plant from the same family. For example, plants that belong to the family Acanthaceae like *A. bicolor* Moon, *A. gangetica* L. T. Anderson and *S. crispus* showed low inhibition while those from the family Falaceae were observed to show a wider range of anti-lipase activity. For example, *A. jiringa* (Jack) I.C Nielsen and *C. cauliflora* have a significant inhibitory effect on lipase while *Leucaena leucocephala* (Lam) Dewit, *O. bancana* (Miq.) Merr., and *P. speciosa* Hassk showed relatively low inhibitions.

In many cases, the pharmacological activities of these plants are attributable to the presence of secondary metabolites such as polyphenols, saponins, tannins, terpenes, flavonoids and alkaloids which are active inhibitors of pancreatic lipase [22,23]. For example, saponins, tannins, alkaloids and flavonoids have been reported in *A. carambola* L. fruits [15], while *A. moluccana* (L.) Willd has been shown to contain bioactive diterpenoids, moluccanic acids, 3,4-secopodocarpane trinorditerpenoids, moluccanic acids, 6,7-dehydromoluccanic acids, and moluccanic acid methyl ester [22]. Therefore, one reason why some of the plant extracts used in the study demonstrated high anti-lipase activity could be due to higher contents of bioactive compounds in their tissues. To date, there have been no reports on the phytochemicals of *A. jiringa* and *C. cauliflora* although there are some reports on their biological activities [24,25].

Apparently plants are not only sources of anti-lipase compounds as 12.2% of the plant extracts screened were found to promote the activity of the enzyme instead (Table 2). Of these, the seeds of one plant, *P. anisum* L., caused 186.5% activation of PPL activity compared to the control. Seven (7) other extracts showed less than 20% activation of lipase activity, and the rest exhibited between 20% to 40% activation. It should be noted that while the leaves of *M. hastata* (L.) Solms inhibited PPL activity, the root enhanced the activity slightly. The therapeutic effects of *P. anisum* have been reported such as being gynaecologic, neurologic and hypothermic, having the ability to delay the onset of picrotoxin-induced seizures in mice, and being a muscle relaxant and anticonvulsant [26,27]. The ethanolic extract of this plant was also reported to contain methylchavicol, eugenol, psedoisoeugenol, anisaidehyde, caffeic acid derivatives, flavonoids, polyacetylene and polyenes as the major constituent compounds.

In this study, an *in vitro* analysis of anti-lipase activity of *C. cauliflora* leaves was conducted in order to confirm its traditional use as an anti-obesity plant. Results obtained show that the EtOAc fraction of the crude methanol extract of the plant was found to be the most active followed by the *n*-BuOH fraction. Bioassay activity-guided fractionation of this fraction by the use of successive column chromatographic methods and hydrophobic Sephadex chromatography have led to the isolation and identification of a flavonoid type compound that is responsible for the inhibition of pancreatic lipase activity. The structure of the compound was elucidated to be kaempferol*-3-O-*rhamnoside by a comparison of one and two-dimensional NMR data which entirely match the previously reported values.

In the search for effective anti-obesity compounds from natural sources, several extracts from plants, and bacterial, fungal, and marine species have been screened in order to find new compounds with pancreatic lipase inhibitory activity. Most of the common compounds that are found in several plants species with anti-lipase activity are polyphenols, saponins and terpenes [28]. For example, licochalcone A, isolated from the ethyl acetate/*n*-hexane fraction of ethyl acetate extract of the roots of *Glycyrrhiza uralensis*, was shown to significantly inhibit the activity of pancreatic lipase at an IC_50_ value of 35 µg/mL [29]. Phenylboronic acid was the potent inhibitor of lipase isolated from *Oryza sativa* [30] while carnosic acid, a diterpine, was the one isolated from the methanolic extract of the leaves of sage, *Salvia officinalis* [31].

Flavonoids such as kaempferol*-3-O-*rhamnoside, which are usually found in the plant kingdom, are widely dispersed and there have been numerous reports on their antioxidant, anti-inflammatory and anti-carcinogenic activities [32]. Flavonoids have been used for the treatment of patients with intolerance to radiation therapy, vascular diseases, liver diseases, and dementia [32]. There exist many pharmacological activities for kaempferol*-3-O-*rhamnoside which are of vital important to health. For example, Nazemiyeh *et al*. [33] reported that this compound has a free radical scavenging ability in animals. This was basically attributed to the existence of a phenolic moiety in the structure. The compound, among other flavonoids, was also found to be active against antibiotic-resistant bacteria [34].

## 4. Experimental

### 4.1. Materials

Medicinal, herbal and aquatic plants that were used in this study were obtained from either the Universiti Putra Malaysia farms (Serdang and Bintulu campuses) or local markets. The plants were identified by the Herbal Unit of Universiti Putra Malaysia. Porcine pancreatic lipase (PPL, Type II), orlistat (N-formyl-L-leucine (1*S*)-1-[(2*S*,3*S*)-3-hexyl-4-oxetanyl]methyldodecyl ester) and gum arabic were purchased from Sigma Aldrich (St. Louis, MO, USA). Other chemicals used in the study were from Merck (Darmstadt, Germany) and were either of laboratory or analytical grade.

### 4.2. Extraction of Plant Metabolites

All plant materials were washed under running tap water before they were cut into smaller pieces and placed in an oven to dry at 45 °C for a minimum of 48 h. Dried samples were ground in a Waring blender (Model 32 BL 80, New Hartford, NY, USA) to obtain a fine powder. Two hundred grams of the ground plant materials were placed in 2 L of 80% methanol, mixed well and steeped for two days at room temperature (~30 °C). The extracts were then filtered through Whatman No. 1 filter paper and the solvent was removed by vacuum evaporation at 42 °C. The dried extracts were stored at −20 °C prior to the analysis; two or three batches of the extracts were obtained depending on the plant sample.

### 4.3. Assay for Pancreatic Lipase Activity

The activity of the enzyme was determined according to the method described previously [9] with some modifications. Instead of measuring the release of radioactive free fatty acids, fatty acids produced during the hydrolysis of substrate was quantified by titration. The substrate was prepared by homogenizing 10% (w/v) refined, bleached and deodorized palm olein in 50 mM Tris-HCl (pH 8.2) buffer solution containing 10% gum arabic. Two milliliters of each plant extract (300 µg/mL dissolved in 5% ethanol at 50 °C) was incubated separately with 2.0 mL of 10 mg/mL PPL solution at 37 °C, 120 rpm for 15 min. Then, 3.0 mL of 50 mM Tris-HCl buffer (pH 8.2), 0.5 mL of 0.5 M CaCl_2_ solution, and 2.5 mL of substrate were added and the mixture incubated under the same conditions (37 °C, 120 rpm) for another 15 min. The reaction was stopped by adding 20 mL of a 1:1 mixture of acetone and ethanol. The resultant mixture was titrated with 50 mM NaOH solution to pH 8.2. PPL inactivated by heating at 100 °C for 5 min was used in the control assay. Percent inhibition, I, was calculated according to the equation below where A is the average volume of NaOH solution needed for enzymatic reaction without an extract/orlistat; a is the average volume of NaOH solution needed for control without an extract/orlistat; B is the average volume of NaOH solution needed for enzymatic reaction with an extract/orlistat; b is the average volume of NaOH solution needed for control with an extract/orlistat. Activation or stimulation of PPL activity is obtained when the value of B is greater than that of A. Results were based on triplicate analysis [9]. 

*I%* = [(*A* – *a*) - (*B* – *b*)] × 100/(*A* – *a*) (1)

### 4.4. Effect of Orlistat on Lipase Activity and Orlistat Equivalent

Since orlistat is a pancreatic lipase inhibitor, its activity against PPL was used to express the relative anti- or pro-lipase activity of plant extracts (orlistat equivalents). The effect of orlistat on PPL activity was evaluated based on the method described above. A 0.5 mg/mL stock solution of orlistat, prepared by dissolving it in 95% isopropanol and making up to 10 mL with 5% isopropanol, was used to prepare a series of solutions (0 to 0.1 µg/mL) to construct a standard curve. The standard curve was rectilinear for the range of concentrations used with y = 924.68x and R^2^ = 0.9937. The anti-lipase activity of the plant extracts was expressed as µg orlistat equiv./mL.

### 4.5. Column Chromatography of EtOAc Fraction of *C. cauliflora* Leaves

Six grams of the EtOAc fraction were subjected to column chromatography with silica gel (Merck Kieselgel 60 Art No. 9385.1000 of particle size 0.040–0.063 mm) normal phase (NP) according to the developed method. The column was prepared by introducing the slurry of adsorbent (189 g) with 100% chloroform (CHCl_3_) into a glass column, which was half filled with the solvent. The packed column (11.6 × 6.5 cm diameter) was conditioned with the mobile phase comprising chloroform–ethyl acetate (CHCl_3_–EtOAc) at 9.5:0.5 ratio. The active EtOAc fraction was dissolved in pure acetone and coated with silica gel in the ratio of (1:1). The resulting slurry was evaporated and then applied on the top of the packed column. The separation of compounds in the mixture was done by eluting with the above solvents of increasing polarity. About 100 mL of the eluant was collected for each fraction. The TLC profile of each fraction was obtained. Each fraction of the sample solution was dotted on the base line of the TLC plate drawn about 1 cm from the bottom of the TLC sheet by means of a fine capillary tube. Profiles of these samples were developed with methanol–water at 1:1 ratio, in which similar fractions were pooled together yielding twenty sub-fractions.

#### Thin Layer Chromatography (TLC)

Analytical TLC was carried out using aluminum sheets of dimensions 40 mm × 80 mm × 0.25 mm coated with silica gel 60 F_254_ (Merck 1.05735). Samples solution obtained from the column chromatography as well as pure compound of the leaves of *C. cauliflora* was spotted or dotted on the base line drawn about 1 cm from the bottom of the TLC sheet by means of a fine capillary tube. The dried sheet was developed in a chromatographic tank saturated with vapor of the mobile phase (1:1 methanol–water) at room temperature (average 35 °C). The spots were then examined under UV light via both long and short wave lengths at 254 and 365 nm respectively. The reagent used for detection was vanillin-sulfuric acid. The acids was utilized to stain the spots and detect the presence of certain type of natural compounds such as higher alcohols, steroids, phenols, flavonoids, and essential oils by spraying the solution on the TLC plate, and then heated at 120 °C. Stained spots indicate the presences of a particular natural product(s).

### 4.6. Dyeing Reagent for TLC

#### Preparation of Vanillin-Sulfuric Acid Solution

An acid solution of vanillin-sulfuric acid was prepared by dissolving 1 g of vanillin crystals in 1 mL of concentrated sulfuric acid and 98 mL of absolute ethanol. The developed TLC plates were sprayed with the solution above, and then heated at 120 °C. The stained spots on the plates indicate the existence of some natural products such as higher alcohols, phenols, essential oils and steroids.

### 4.7. Instrumentation

#### Nuclear Magnetic Resonance (NMR)

^1^H-Nuclear magnetic resonance (^1^H-NMR) spectra were measured and recorded in deuterated methanol (CD_3_OD) by means of a Varian FT NMR 500 MHz spectrometer. Chemical shifts (δ) were recorded in parts per million (ppm) relative to the tetramethylsilane (TMS) signal used as internal standard. The signals were illustrated in terms of chemical shift with proper abbreviations for multiplicities and are reported as *s* (singlet), *d* (doublet), and *t* (triplet). Moreover, ^13^C-Nuclear Magnetic Resonance (^13^C-NMR) with off resonance decoupling heteronuclear multiple bond coherence (HBMC), heteronuclear single quantum correlation (HSQC) and correlation spectroscopy (COSY) were all recorded on the Varian FT NMR 500 MHz spectrometer.

## 5. Conclusions

This study shows that while many plants are capable of inhibiting lipase activity, and therefore, concur with many reports on anti-lipase plants, there are some those actually stimulate the activity of the enzyme. The remarkable inhibitory activity (greater than 80%) of some of these plant extracts indicates their potential use as important and convenient sources of anti-obesity agents. One of these plants was *C. cauliflora* and the leaves were used to isolate and identify the structure of its anti-lipase compound. It was established that kaempferol 3-*O*-rhamnoside is a new anti-lipase inhibitor isolated from the leaves extract of *C. cauliflora*. The finding provides evidence for anecdotal use of the plant as an anti-obesity agent and may help in development of a natural anti-obesity agent. The study also indicates that the inclusion of *P. anisum* L. in the diet may have the opposite effect as the extract from the seeds of this plant actually stimulates pancreatic lipase activity. On the other hand, it may be the spice that is suitable as an ingredient in concoctions for those wanting a weight increase.
